# The Formation of a Unique 2D Isonicotinate Polymer Driven by Cu(II) Aerobic Oxidation

**DOI:** 10.3390/ma16103724

**Published:** 2023-05-14

**Authors:** Francisco Sánchez-Férez, Teresa Calvet, Mercè Font-Bardia, Josefina Pons

**Affiliations:** 1 Departament de Química, Universitat Autònoma de Barcelona, 08193 Bellaterra, Spain; francisco.sanchez.ferez@uab.cat; 2 Departament de Mineralogia, Petrologia i Geologia Aplicada, Universitat de Barcelona, Martí i Franquès s/n, 08028 Barcelona, Spain; 3 Unitat de Difracció de Raig-X, Centres Científics i Tecnològics de la Universitat de Barcelona (CCiTUB), Universitat de Barcelona, Solé i Sabarís, 1-3, 08028 Barcelona, Spain

**Keywords:** Cu(II) polymers, 2D materials, aerobic oxidation, isonicotinic acid, 4-acetylpyridine

## Abstract

The isolation and structural characterization of a unique Cu(II) isonicotinate (ina) material with 4-acetylpyridine (4-acpy) is provided. The formation of [Cu(ina)_2_(4-acpy)]_n_ (**1**) is triggered by the Cu(II) aerobic oxidation of 4-acpy using O_2_. This gradual formation of ina led to its restrained incorporation and hindered the full displacement of 4-acpy. As a result, **1** is the first example of a 2D layer assembled by an ina ligand capped by a monodentate pyridine ligand. The Cu(II)-mediated aerobic oxidation with O_2_ was previously demonstrated for aryl methyl ketones, but we extend the applicability of this methodology to heteroaromatic rings, which has not been tested so far. The formation of ina has been identified by ^1^H NMR, thus demonstrating the feasible but strained formation of ina from 4-acpy in the mild conditions from which **1** was obtained.

## 1. Introduction

The discovery of graphene foregrounded the outbreak of 2D materials, which was triggered by their fascinating electrochemical, mechanical, and optical properties. Their better performance over 0D and 1D materials fostered the rapid development of 2D-based technologies for electrochemical devices, renewable energy storage, and production or for catalysis. Especially in these fields, the aim is, *per se*, to encounter clean, renewable, and inexpensive sources, so Cu(II)-based materials were expected to be one of the most promising candidates since Cu(II) combines high natural abundance with low toxicity [1].

Within this frame, our group has previously reported the synthesis and characterization of Cu(II) complexes with carboxylic acids and pyridine derivatives [2,3,4]. During the assays to further extend this research with 4-acetylpyridine (4-acpy), we performed its reaction with Cu(NO_3_)_2_·3H_2_O in acetonitrile (ACN) as a solvent. From this reaction, the serendipitous formation of single crystals, which were isolated and characterized, revealed the formation of an isonicotinate (ina) ligand, which further coordinated to the Cu(II) center and fostered the formation of complex [Cu(ina)_2_(4-acpy)]_n_ (**1**). Therefore, in this contribution, we present the serendipitous formation of a Cu(II) isonicotinate (ina) material starting from Cu(NO_3_)_2_·H_2_O and 4-acpy.

Interestingly, the elucidation of its crystal structure revealed the arrangement of a 2D isonicotinate material. Indeed, isonicotinic acid (ina) has been vastly employed as an archetypal linker for the construction of extended networks. In particular, Cu(II) isonicotinates have presented from 0D to mostly 3D structures, benefiting from the great variety of coordination modes of ina ranging from monodentate to bridging. Within this plethora of available structures, Cu(II) ions mainly present square pyramidal geometry and lead to the assembly of highly stable 3D nets [5] featured by [Cu(ina)_2_]_n_ [6]. This tendency of forming 3D nets is only broken either by introducing polydentate chelate or bridging ligands that are able to compete with the coordination of ina, which lowers the dimensionality to 1D [7] or 2D materials [8,9], or the isolated example of a 0D structure by combining ina with a chelate linker, coordinated chloride ions, and bulky counterions [10]. Therefore, the dimensionality of Cu isonicotinates is biased towards 3D nets, and no previous examples have been reported with monodentate pyridine derivatives, which are probably triggered by its lower coordination ability. Additionally, hydrothermal conditions promoted the formation of mixed valent Cu(I)-Cu(II) isonicotinates rather than guiding a tunable dimensionality [11]. Therefore, the slow formation of ina, in combination with the excess 4-acpy, provided access to a network that has not been achieved by direct self-assembly [6].

Since the formation of ina seemed to be triggered by Cu(II) with molecular oxygen as the oxidizing agent, we tried to demonstrate the feasible formation of ina from 4-acpy in the mentioned conditions. Within the palette of available first-row transition metals, Cu(II) was already known to promote C-N, -S, and -O bond formation, avoiding air moisture sensitivity and providing elevated functional group tolerance [12]. However, despite the promising advantages, the early stages of this research with limited examples hitherto found triggered the use of Pd(II) with the consequent overshadowing of Cu(II). Conversely, during the last few decades, the course of Cu(II) research was shifted by the buoyant results that emerged.

To date, Cu(II) catalytic systems have shown the ability to oxidize a plethora of organic substrates using molecular oxygen. Among the major findings of special mention are the benzylic oxidation to alcohols [13], or mixtures of alcohols and ketones [14]; the oxidation of alkanes to alcohols and ketones [15]; the oxidation of alcohols to aldehydes [16,17], or to mixtures of aldehydes and ketones [18], the oxidation of primary and secondary alcohols to aldehydes and ketones [19,20]; the oxidation of phenols to ortho-quinones, biphenols, or benzoxepines [21], and the oxidation of amines to imines and nitriles [22], not to mention Baeyer–Villiger oxidation [23]. Indeed, the catalytic oxidation of aryl methyl ketones to mixtures of aldehydes and carboxylic acids using molecular oxygen as an oxidant was tested several years ago [24], but recently, some methodologies for the isolation of aldehydes [25], esters [26], and carboxylic acids [27,28] have been published.

Thus, we present the serendipitous formation of **1** promoted by the slow catalytic oxidation, using molecular oxygen, of the acetyl group of 4-acpy to the carboxylate functionality in ina. The single-crystal X-ray diffraction of **1** displayed a 2D coordination polymer assembled by ina and 4-acpy ligands, which was subsequently characterized by EA and FTIR-ATR spectroscopy. Additionally, we demonstrated the feasibility of the formation of ina from 4-acpy, and we tried to obtain some insights into the effect of changing the synthetic conditions (changing the Cu(II) salt, the solvent, and the O_2_ pressure) of the resulting product. These experiments were traced by ^1^H NMR spectroscopy.

## 2. Experimental Section

### 2.1. Materials and General Methods

Copper(II) nitrate trihydrate (Cu(NO_3_)_2_·3H_2_O), copper(II) acetate hydrate (Cu(OAc)_2_·H_2_O), and 4-acetylpyridine (4-acpy) as reagents and acetonitrile (ACN) and N,N-dimethylformamide (DMF) as solvents were purchased from Sigma-Aldrich. In addition, deuterated ACN (ACN-*d*_3_) and DMF (DMF-*d*_7_) were used for the ^1^H NMR experiments. Reagents and solvents were used as received without further purification. Elemental analysis (C, H, and N) was measured in a Euro Vector 3100 apparatus. FTIR-ATR spectrum was measured in a diamond window in the range from 4000 to 500 cm^−1^ in a Tensor 27 (Bruker) spectrometer equipped with an attenuated total reflectance (ATR) accessory (model MKII Golden Gate). ^1^H NMR spectra were acquired using a Bruker Ascend 400 MHz spectrometer either in ACN-*d_3_* or DMF-*d*_7_ at room temperature. All the chemical shifts (δ) are given in ppm.

#### 2.1.1. Synthesis of [Cu(ina)_2_(4-acpy]_n_ (1)

To an ACN solution (6 mL) of Cu(NO_3_)_2_·3H_2_O (12.0 mg, 0.050 mmol), liquid 4-acpy (23 µL, 0.317 mmol) was added. The reaction was stirred under reflux for 16 h and then was transferred to a vial and left to slowly evaporate for 1 month. After this period, suitable green crystals of **1** were grown.

**1**. Elemental Analysis calcd(%) for C_19_H_15_CuN_3_O_5_ (428.88 g/mol): C 53.21; H 3.53; N 9.80; found: C 52.42; H 3.29; N 9.52. FTIR-ATR (wavenumber, cm^−1^): 3072(w), 2908(w), 2795(w), 1695(m), 1631(m), 1608(w), 1590(s), 1500(m), 1489(m), 1436(s), 1384(s), 1360(s), 1323(m), 1255(s), 1239(s), 1216(m), 1170(m), 1112(m), 1074(m), 1033(s), 964(w), 933(m), 919(s), 883(m), 840(w), 831(w), 819(m), 804(s), 774(s), 767(s), 722(m), 682(m), 668(s), 590(s), 554(m).

#### 2.1.2. X-ray Crystallographic Data

A green prism-like specimen of **1** was used for the crystallographic data collection. The X-ray intensity data were measured on a D8 Venture system equipped with a multilayer monochromator and a Mo microfocus. The frames were integrated with the Bruker SAINT software package, using a narrow-frame algorithm. The integration of the data with a 0.70 Å resolution gave an average redundancy of 7.101, a completeness of 99.7%, and an R_sig_ of 4.05%. From this integration, 3135 (82.33%) independent reflections were greater than 2σ(|*F*|^2^).

The structure was solved and refined using the Bruker SHELXTL Software Package (version-2018/3) [29]. The final cell constants and volume are based upon the refinement of the XYZ-centroids of reflections above 20 σ(I). Data were corrected for absorption effects using the multi-scan method (SADABS). Crystal data and relevant details of structure refinement are reported in Table 1. The entire X-ray data of **1** can be found via the CCDC in .cif format, using the code 2252448. The X-ray structure was worked with Mercury 4.3.1 software, and molecular graphics were rendered using the POV-Ray image package [30]. Color codes used for the molecular graphics are orange roughy (Cu), red (O), light blue (N), grey (C) and white (H).

The geometry evaluation of the Cu(II) center in the complex has been performed using version 2.1 of SHAPE [31] software, which is based on the low continuous-shape measure (CShM) value *S* [32]. It is a generalizable structural descriptor used to quantitatively evaluate distortion in terms of symmetry and distance from any ideal geometry. The corresponding atomic coordinates have been directly extracted from .cif data and the *S* values have been computed for any potential geometric accommodation within the corresponding coordination number five: vOC-5 = vacant octahedron; TBPY-5 = trigonal bipyramid; SPY-5 = square pyramid; JTBPY-5 = Johnson trigonal bipyramid.

## 3. Results and Discussion

### 3.1. Synthesis and General Characterization

**Scheme 1:**
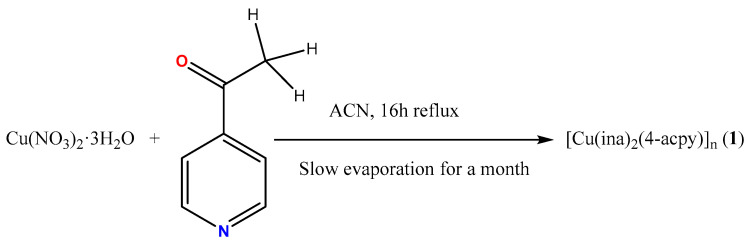
Ouline of the synthetic conditions to the formation of **1**.

The complex [Cu(ina)_2_(4-acpy)]_n_ was synthesized by mixing in ACN the Cu(NO_3_)_2_·3H_2_O and the 4-acpy ligand in a 1:6 molar ratio, stirring under reflux, and then leaving it to stand for a month (Scheme 1). During this period, suitable green crystals of **1** were grown. The product was characterized by elemental analysis (EA), FTIR-ATR spectroscopy, and single-crystal X-ray diffraction.

The EA of **1** agrees with the proposed formula. The displayed signals within the FTIR-ATR spectrum are attributable to vibrations from both ligands, ina and 4-acpy (S.I: Figure S1). The most characteristic vibrations from 4-acpy are found at 2908 and 2795 cm^−1^, assigned to [ν(C-H)]_al_, and at 1695 cm^−1^ from the [ν(C=O)] of the acetyl group. Vibrations over 3000 cm^−1^ belong to [ν(C-H)]_ar_ from the aromatic rings of both ligands, whereas signals from [ν(C=C/C=N)]_al_ can be found between 1631 and 1436 cm^−1^. The formation and further coordination of the ina ligand cause the raising of vibrations from the carboxylate functionality. Bands at 1590 and 1384 cm^−1^ have been attributed to [ν(COO)]_as_ and [ν(COO)]_s_, respectively. Indeed, the coordination mode of the carboxylate in the Cu(II) complex can be inferred from the difference between these bands (referred to as the Δ value) [33,34]. In **1**, the Δ of 206 cm^−1^ suggests a monodentate coordination mode. Additional bands from the aromatic rings attributed to [δ(C=C/C=N)] between 1360 and 1239 cm^−1^, [δ(C-H)]_ip_ at 1033 cm^−1^, and [δ(C-H)]_oop_ at 774 cm^−1^ have also been identified [35].

### 3.2. Crystal Structure of [Cu(ina)_2_(4-acpy)]_n_ (1)

**Figure 1:**
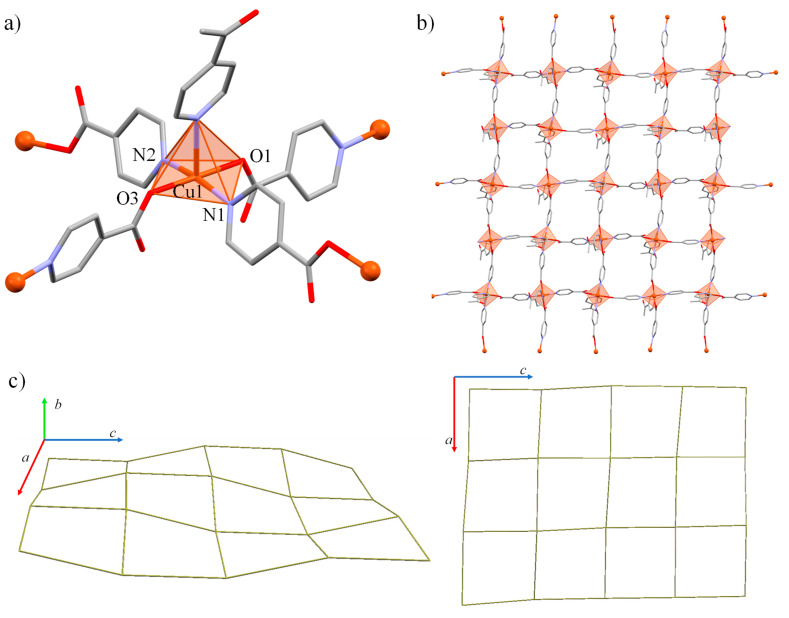
Representation of (**a**) the molecular structure of complex **1** with atom labeling, (**b**) the pairing of 4-acpy within the plane displaying an upwards or downwards disposition, and (**c**) the sql topology.

[Cu(ina)_2_(4-acpy)]_n_ (1) crystallizes in the orthorhombic Pbca space group and contains Cu(II) centers bearing a [CuO_2_N_3_] *core* with a distorted square pyramidal geometry (*S* = 0.942 for SPY, 4.456 for TBPY, 1.006 for vOC, and 7.824 for JTBPY) [31,36]. These units are composed of four bridging (µ_2_:ƞ^1^:ƞ^1^-) ina ligands and a monodentate (µ_1_:ƞ^1^-) 4-acpy ligand (Figure 1a), which are orthogonally connected by ina ligands, being 4-acpy sequenced in pairs by pointing upwards and downwards along each chain (Figure 1b). This linkage results in the formation of 2D layers along the (030) plane holding an sql topology (Figure 1c). Selected bond lengths and angles are provided in Table 2. The equatorial Cu-O and Cu-N bond lengths from isonicotinate ligand in **1** are comparable to those extracted from [Cu(ina)_2_]_n_ (Cu-O, 1.962(3)–2.306(2) Å and Cu-N, 2.010(5)-2.026(2) Å) [6]. Instead, the apical site is occupied by 4-acpy with an elongated Cu-N bond length of 2.351(3) Å, which is probably driven by a marked Jahn–Teller effect [37]. This length is surprisingly long compared to the Cu(II) complexes with 4-acpy reported to date (Cu-N 2.016–2.198(2) Å) [38,39,40,41,42,43].

**Figure 2:**
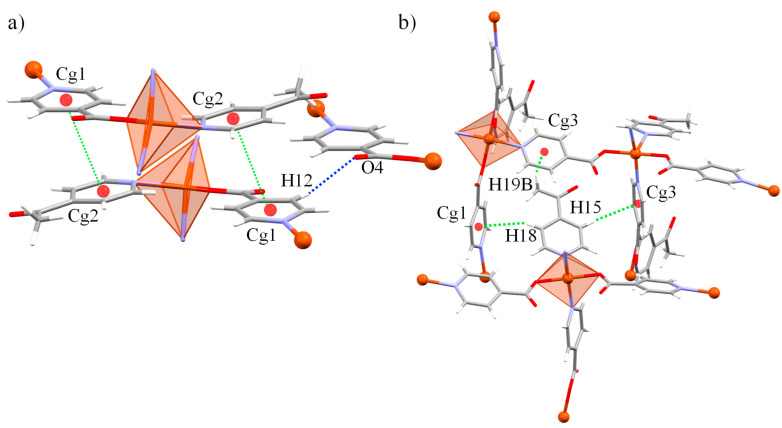
Representation of the intermolecular (**a**) π···π and C-H···O or (**b**) C-H···π interactions present in complex **1**. Dashed blue lines stand for C-H··· O interactions, whereas dashed green lines refer to C-H···π and π···π interactions.

These layers are assembled by π···π and C-H···O and C-H···π interactions resulting in a 3D supramolecular net. The aromatic ring between ina and 4-acpy ligands are stacked at 3.868(2) Å supported by C-H···O interactions between the uncoordinated *O* carboxylate and the *ortho*-H of a vicinal ina ligand (Figure 2a). Furthermore, each 4-acpy is embedded into a pocket of ina ligands and displays three C-H···π interactions (Figure 2b).

### 3.3. Catalytic Conversion

As previously mentioned, the aerobic oxidation of aromatic methyl ketones to carboxylic acids using Cu(II) as the catalyst has been previously reported, but no examples have been found using the analogous heteroaromatic molecules. After the obtention of complex **1**, we tried to follow the conversion from 4-acpy to ina by ^1^H NMR spectroscopy. To this aim, we tested the oxidation of 4-acpy to ina, modifying the precursor, the solvent, and the O_2_ pressure. These results have been summarized in Table 3.

As a general procedure adapted from [27], 0.100 mmol of the Cu(II) salt (24.16 mg of Cu(NO_3_)_2_·3H_2_O or 19.96 mg of Cu(OAc)_2_·H_2_O) and 0.6 mmol of 4-acpy (68 µL) were placed in a vial and dissolved in either 2 mL of ACN or DMF, and the resulting dark blue solution was degassed. Then, the vials were filled with 2.1 bars of O_2_ pressure, sealed, and put in the furnace at 120 °C for 18 h. Then, the reaction crude was dried and dissolved in DMF-*d*_7_ or ACN-*d*_3_ for the ^1^H NMR experiments.

First, aiming to demonstrate the feasibility of the catalytic conversion from 4-acpy to ina under similar experimental conditions from which **1** was isolated, the reaction between Cu(NO_3_)_2_·3H_2_O and 4-acpy was carried out in ACN under autogenous pressure at 120 °C for 18 h. Reactions were conducted using a closed vessel based on previous examples, evincing a boost in the reaction rate to achieve faster conversion [27].

**Figure 3:**
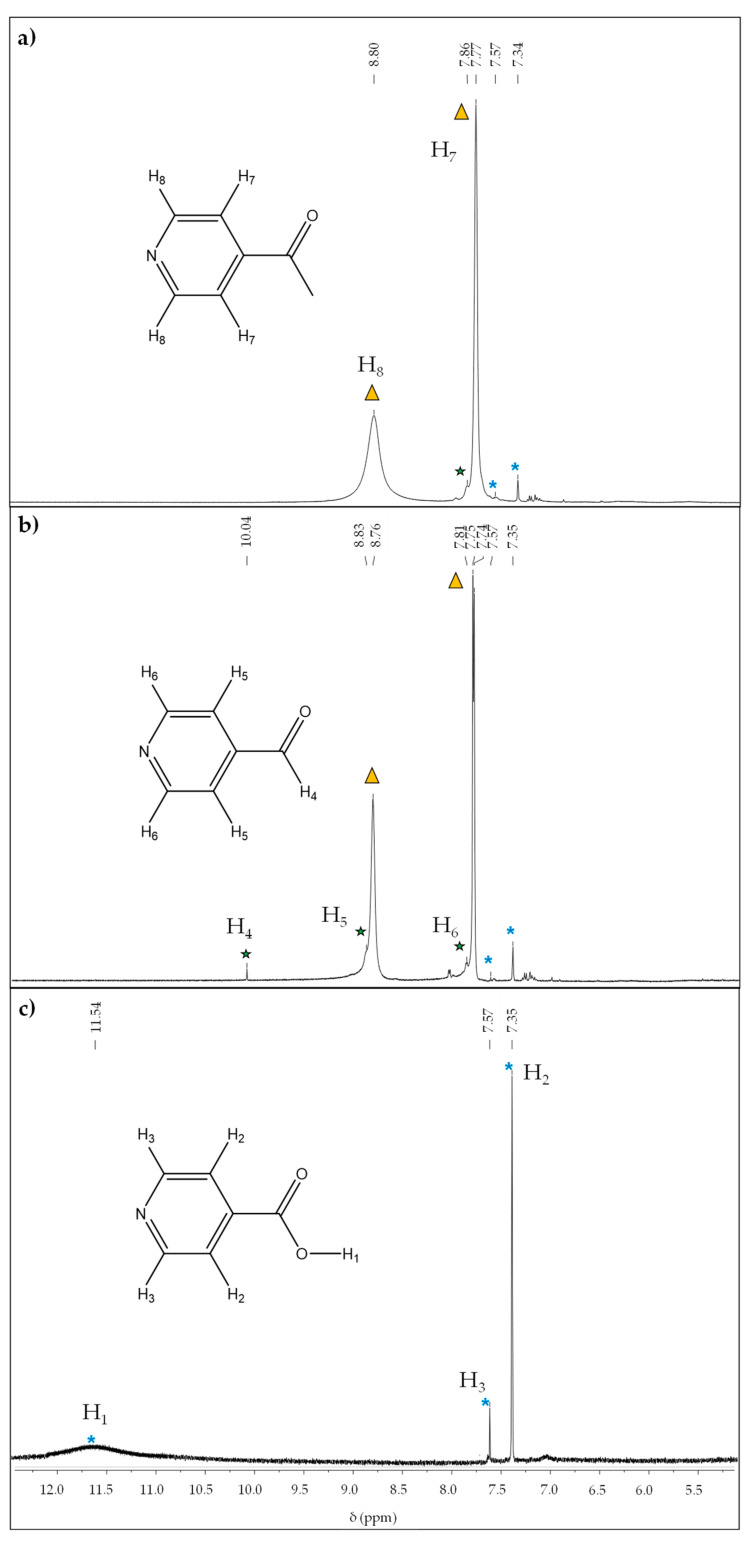
^1^H NMR spectra in ACN-*d*_3_ of the catalytic assays: (**a**) using Cu(NO_3_)_2_·3H_2_O in ACN without O_2_ pressure for 18 h at 120 °C; (**b**) using Cu(OAc)_2_·H_2_O in ACN at 2.1 bars of O_2_ pressure for 18 h at 120 °C; (**c**) using Cu(NO_3_)_2_·3H_2_O in ACN at 2.1 bars of O_2_ pressure for 18 h at 120 °C. Peaks belonging to 4-fopy are identified with green-filled stars, those belonging to 4-acpy are identified with yellow-filled triangles, and those belonging to ina are identified with light blue asterisks.

The ^1^H NMR in ACN-*d*_3_ of the resulting products revealed the recovery of 4-acpy and the mixtures of the corresponding aldehyde (4-formylpyridine, 4-fopy) and ina, therefore verifying that the Cu(II) catalytic oxidation of 4-acpy to ina is attainable (Figure 3a). Then, to further extend these results, we scrutinized different synthetic conditions to evaluate this process. To this end, two different solvents were employed: ACN and DMF. It should be mentioned that reactions performed in DMF resulted in the recovery of 4-acpy without any trace of 4-fopy nor ina, regardless of the Cu(II) precursor or the addition of 2.1 bars of O_2_ pressure (S.I: Figure S2). In addition, the use of Cu(OAc)_2_·H_2_O in ACN leads to the recovery of 4-acpy and mixtures of 4-fopy and ina (Figure 3b), yielding a similar result to that of Cu(NO_3_)_2_·3H_2_O without O_2_ pressure.

**Scheme 2:**
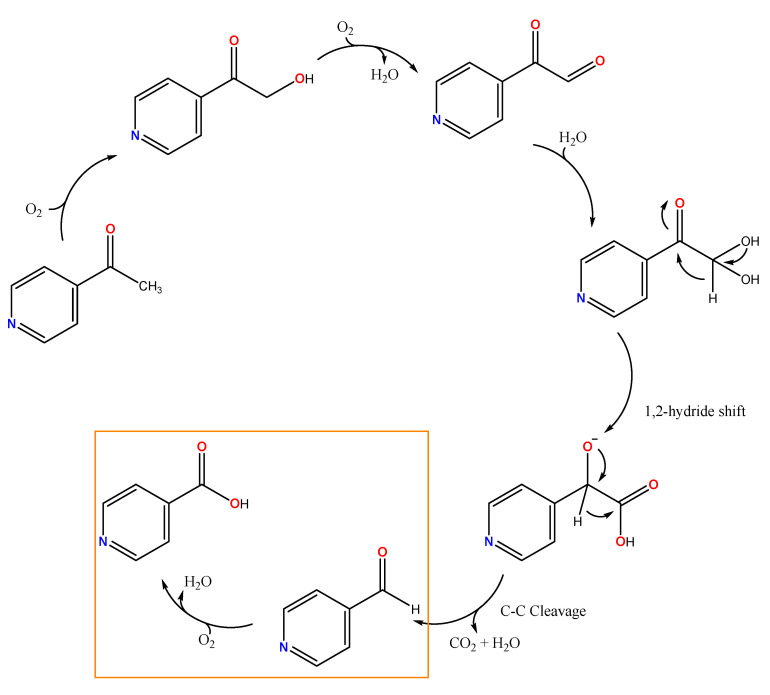
Mechanistic pathway for the conversion of 4-acpy into ina or 4-fopy. The orange square highlights the two products identified: ina and 4-fopy. Adapted from [28].

Interestingly, employing Cu(NO_3_)_2_·3H_2_O and 4-acpy in ACN at 2.1 bars of O_2_ pressure and heating up to 120 °C for 18h were found to be the best experimental conditions since only ina has been identified and no traces of additional products are present (Figure 3c). This is in agreement with the previous results with aromatic methyl ketones [27]. It should be mentioned that, from this reaction, a blue powder was isolated. After the filtration of the reaction crude and washing with 10 mL of DMF, the remaining solid was identified as [Cu(ina)_2_(H_2_O]_n_ (S.I: Figure S3). This agrees with the two requirements needed to achieve the formation of **1**: the gradual formation of the ina ligand and a sufficient amount of 4-acpy that remains unreacted. Thus, it is reasonable to suggest that the heteroaromatic analogue follows the same catalytic pathway [28], and the presence of 4-fopy is due to the incomplete conversion of 4-acpy into ina (Scheme 2).

Therefore, Cu(NO_3_)_2_·3H_2_O can oxidize 4-acpy to ina even without O_2_ pressure, despite having the worst performance. Among the palette of oxidative reactions from methyl ketones with Cu(II), α-oxygenation to carboxylic acids and C-C bond cleavage to aldehydes can occur in similar conditions. The outcome is mainly biased by the strong dependence of the catalytic performance on the Cu(II) counterion. In this case, and as previous catalytic studies have already noticed, the use of Cu(OAc)_2_·H_2_O as the catalyst led to small quantities of the corresponding aldehyde [25]. Similarly, the absence of O_2_ pressure with Cu(NO_3_)_2_·3H_2_O also yielded mixtures of 4-fopy and ina, which reflects the need for O_2_ to boost the transformation into ina.

It should be noted that the decreased catalytic activity from Cu(OAc)_2_·H_2_O with respect to Cu(NO_3_)_2_·3H_2_O has already been reported and can be attributed to the decreased availability of Cu(II) ions that remain coordinated to the acetate ions. Similarly, the conversion of 4-acpy can be hindered by the coordination to the Cu(II) ions in contrast to aryl methyl ketones.

## 4. Conclusions

We reported, for the first time, the assembly of a Cu(II) isonicotinate bearing a 2D layered structure holding a monodentate pyridine derivative, the 4-acpy ligand. The formation of ina has been traced back, resulting in the identification of the Cu(II) catalytic oxidation of 4-acpy using dioxygen as the oxidant. Thus, we extended the covering of the Cu(II)-driven oxidation of methyl ketones to a heteroaromatic molecule, suggesting an analogous pathway to the one followed by its aryl counterparts. The use of Cu(NO_3_)_2_·3H_2_O without O_2_ pressure or Cu(OAc)_2_·H_2_O at 2.1 bars of O_2_ provided a less efficient conversion, leaving unreacted 4-acpy, which probably drove the arrangement of **1**. Similarly to the analogous reactions with aryl methyl ketones, the best results were garnered using ACN under an O_2_ pressure of 2.1 bars at 120 °C for 18 h, from which ina was isolated. This improved the conversion promoted by the formation of [Cu(ina)_2_(H_2_O]_n_ that rapidly precipitated. Therefore, the remarkable difference in the conversion after changing the Cu(II) precursor demonstrates the significant effect of the counterion. Furthermore, the assays in DMF did not bring any evidence of 4-fopy nor ina ligand formation regardless of the employed Cu(II) salt. Therefore, it seemed that the catalytic performance of Cu(II) in α-oxygenation as well as in the C-C bond cleavage of heteroaromatic methyl ketones behaves similarly to their aryl analogues. Then, the formation of mixtures with 4-fopy when modifying the synthetic conditions is also provided probably as a result of incomplete conversion. It, therefore, appears that this progressive formation of ina, combined with the excess of 4-acpy, enabled its gradual nucleation and growth, granting the formation of suitable crystals for the X-ray diffraction of compound **1**.

## Figures and Tables

**Table 1:** X-ray crystallographic data of complex **1**.

Sample	1
Empirical Formula	C_19_H_15_CuN_3_O_5_
Formula weight	428.88
T (K)	254 (2)
Wavelength (Å)	0.71073
System, space group	Orthorhombic, Pbca
Unit cell dimensions	
a (Å)	12.6673 (11)
b (Å)	11.7649 (9)
c (Å)	24.118 (2)
α (°)	90
β (°)	90
γ (°)	90
V (Å^3^)	3594.4 (5)
Z	8
D_calc_ (mg/m^3^)	1.585
µ (mm^−1^)	1.253
F (000)	1752
Crystal size (mm^−3^)	0.180 × 0.173 × 0.076
hkl ranges	−16 ≤ h ≤ 16 −14 ≤ k ≤ 14 −30 ≤ l ≤ 30
θ range (°)	2.332 to 26.747
Reflections collected/unique/[R_int_]	27,042/3808/0.0405
Completeness to θ (%)	99.6
Absorption correction	Semi-empirical from equivalents
Refinement method	Full-matrix least-squares on |F|^2^
Data/restraints/parameters	3808/1/229
Goodness-of-fit on F^2^	1.034
Final R indices [I>2σ(I)]	R_1_ = 0.0525, wR_2_ = 0.1293
R indices (all data)	R_1_ = 0.0662, wR_2_ = 0.1397
Extinction coefficient	n/a
Largest diff-peak and hole (e. Å^−3^)	1.037 and −0.914

**Table 2:** Bond lengths, angles, and intermolecular interactions in complex **1**.

Bond lengths					
Cu(1)-O(1)		1.949(3)		Cu(1)-N(1)#2	2.038(3)
Cu(1)-O(3)		1.957(2)		Cu(1)-N(3)	2.351(3)
Cu(1)-N(2)#1		2.036(3)			
Bond Angles					
O(1)-Cu(1)-O(3)		179.76(12)		N(2)#1-Cu(1)-N(1)#2	167.80(13)
O(1)-Cu(1)-N(2)#1		88.25(12)		O(1)-Cu(1)-N(3)	90.49(12)
O(3)-Cu(1)-N(2)#1		91.92(11)		O(3)-Cu(1)-N(3)	89.32(11)
O(1)-Cu(1)-N(1)#2		89.66(12)		N(2)#1-Cu(1)-N(3)	99.33(12)
O(3)-Cu(1)-N(1)#2		90.21(12)		N(1)#2-Cu(1)-N(3)	92.71(13)
π···π interactions					
Cg(I)···Cg(J)	Cg···Cg ^a^	A ^b^	β, γ ^c^	Cg(I)_Perp, Cg(J)_Perp ^d^	Slippage ^e^
Cg(1)···Cg(2)	3.868(2)	6.7(2)	18.3, 24.6	3.5170(18), 3.6727(17)	1.213
C-H···π interactions					
C-H···Cg(J)	H···Cg(J) ^f^	H-Perp ^g^	Γ ^c^	X···Cg(J) ^h^	X-H, π ^i^
C18-H18···Cg(1)	2.98	2.87	15.70	3.849(5)	74
C15-H15···Cg(3)	2.96	2.70	23.86	3.777(5)	68
C19-H19B···Cg(3)	2.92	2.87	10.47	3.702(7)	59

#1 x + 1/2, −y + 3/2, −z + 1; #2 x − 1/2, y, −z + 1/2; ^a^ Cg···Cg = distance between ring centroids given in Å; ^a^ Cg···Cg = distance between ring centroids (Å); ^b^ α= dihedral angle between planes I and J (°); ^c^ offset angles: β = angle Cg(I)-Cg(J) and normal to plane I (°), and γ = angle Cg(I)-Cg(J) and normal to plane J (°) (β = γ, when α = 0); ^d^ perpendicular distance (Å) of Cg(I) on plane J and perpendicular distance (Å) of Cg(J) on plane I (equal when α = 0); ^e^ slippage = horizontal displacement or slippage between Cg(I) and Cg(J) (equal for both centroids when α = 0); Cg(1) = N2-C8-C9-C10-C11-C12; Cg(2) = N3-C14-C15-C16-C17-C18; Cg(3) = N1-C2-C3-C4-C5-C6.; ^f^ distance between H atom and ring centroid J (Å); ^g^ perpendicular distance of H to ring plane J; ^h^ distance of X to ring centroid J (Å); ^i^ angle of the X-H bond with the Cg(J) plane.

**Table 3:**
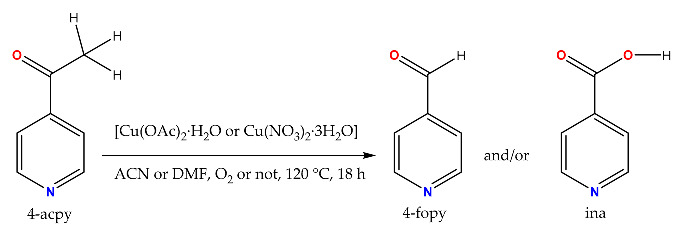
Summary of the results and conditions employed for the catalytic conversion of 4-acpy to ina and/or 4-fopy. Outline of the conversion with the tested conditions.

Precursor	Quantity (mmol)	Solvent	O_2_ Pressure (bar)	Product
Cu(NO_3_)_2_·3H_2_O	24.16 mg (0.1)	DMF	atm	n.r.
		ACN	atm	4-fopy + ina
		DMF	2.1	n.r.
		ACN	2.1	ina
Cu(OAc)_2_·H_2_O	19.96 mg (0.1)	DMF	2.1	n.r.
		ACN	2.1	4-fopy + ina

Experimental conditions: 2 mL of the corresponding solvent at 120 °C for 18 h using 68 µL of 4-acpy. n.r. = no reaction; ACN = acetonitrile; DMF = N,N-dimethylformamide.

## Data Availability

All the data from this research has been included in this manuscript and supporting information.

## Supplementary Materials

The following supporting information can be downloaded at https://www.mdpi.com/article/10.3390/ma16103724/s1. Figure S1: FTIR-ATR spectrum of compound **1**. Figure S2: ^1^H NMR spectra in DMF-*d*_6_ of the catalytic assays using (a) Cu(NO_3_)_2_·2H_2_O in DMF at 2.1 bars of O_2_ pressure for 18 h at 120 °C and (b) Cu(OAc)_2_·H_2_O in DMF at 2.1 bars of O_2_ pressure for 18 h at 120 °C. Figure S3: FTIR-ATR spectrum of compound [Cu(ina)_2_(H_2_O)]_n_.
