# Supplementary material for: The Formation of a Unique 2D Isonicotinate Polymer Driven by Cu(II) Aerobic Oxidation
Source: Materials (Basel). 2023 May 14;16(10):3724. doi: 10.3390/ma16103724 (PMC10221464; doi:10.3390/ma16103724)
Supplement: Supplementary file 1 [file materials-16-03724-s001.zip › materials-2346322-supplementary.pdf]

Supporting Information

The formation of a unique 2D isonicotinate polymer driven by Cu(II) aerobic oxidation

Francisco Sánchez-Férez [a], Teresa Calvet [b], Mercè Font-Bardia [c], and Josefina Pons [a,]*

[a]Departament de Química, Universitat Autònoma de Barcelona, 08193-Bellaterra, Barcelona, Spain

[b]Departament de Mineralogia, Petrologia i Geologia Aplicada, Universitat de Barcelona, Martí i Franquès s/n, 08028 Barcelona, Spain

[c]Unitat de Difracció de Raig-X, Centres Científics i Tecnològics de la Universitat de Barcelona (CCiTUB), Universitat de Barcelona, Solé i Sabarís, 1-3, 08028 Barcelona, Spain

*Corresponding author E-mail: josefina.pons@uab.es

FTIR-ATR and ^{1}H NMR spectrocopies

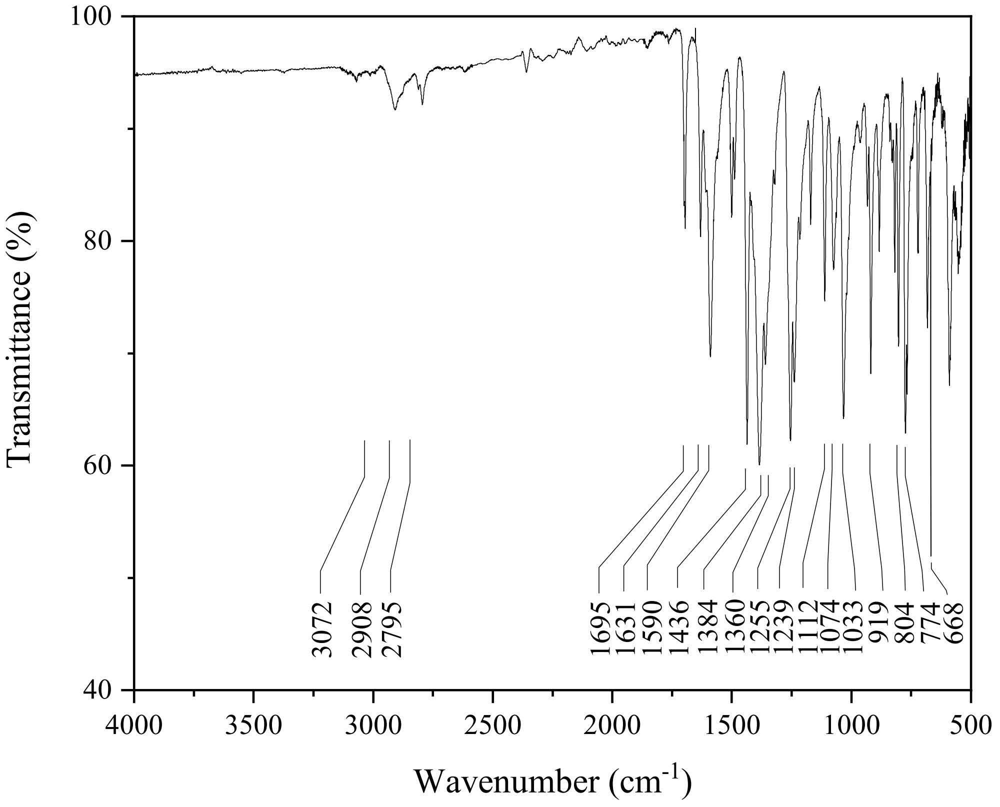

Figure S1. FTIR-ATR spectrum of compound **1**.

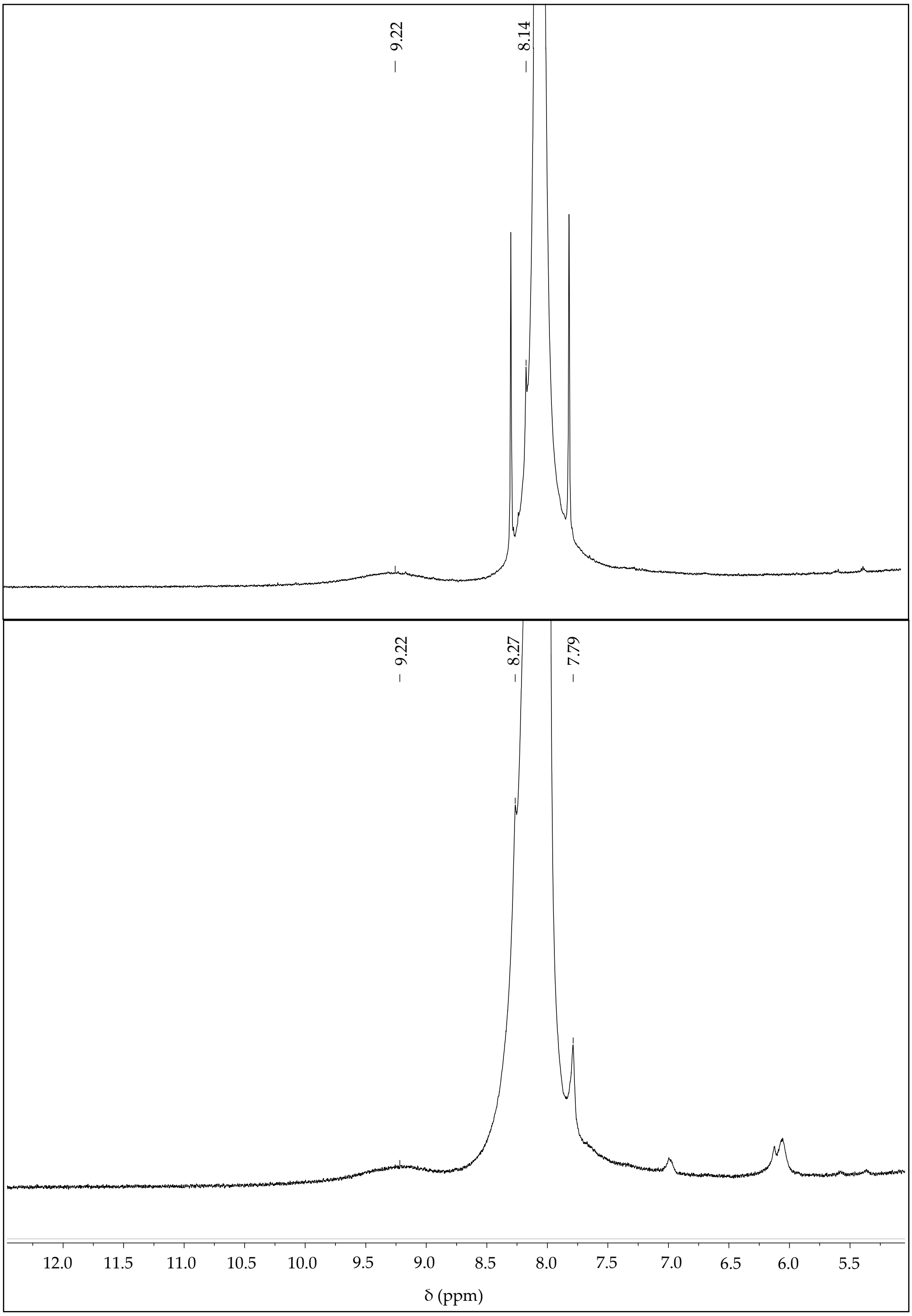

Figure S2. ^{1}H NMR spectra in DMF-d_6 of the catalytic assays. a) using $Cu(NO_3)_2{\cdot}3H_2O$ in DMF at 2.1 bars of O_2 pressure for 18 h at 120 °C, and; b) using $Cu(OAc)_2{\cdot}H_2O$ in DMF at 2.1 bars of O_2 pressure for 18 h at 120 °C.

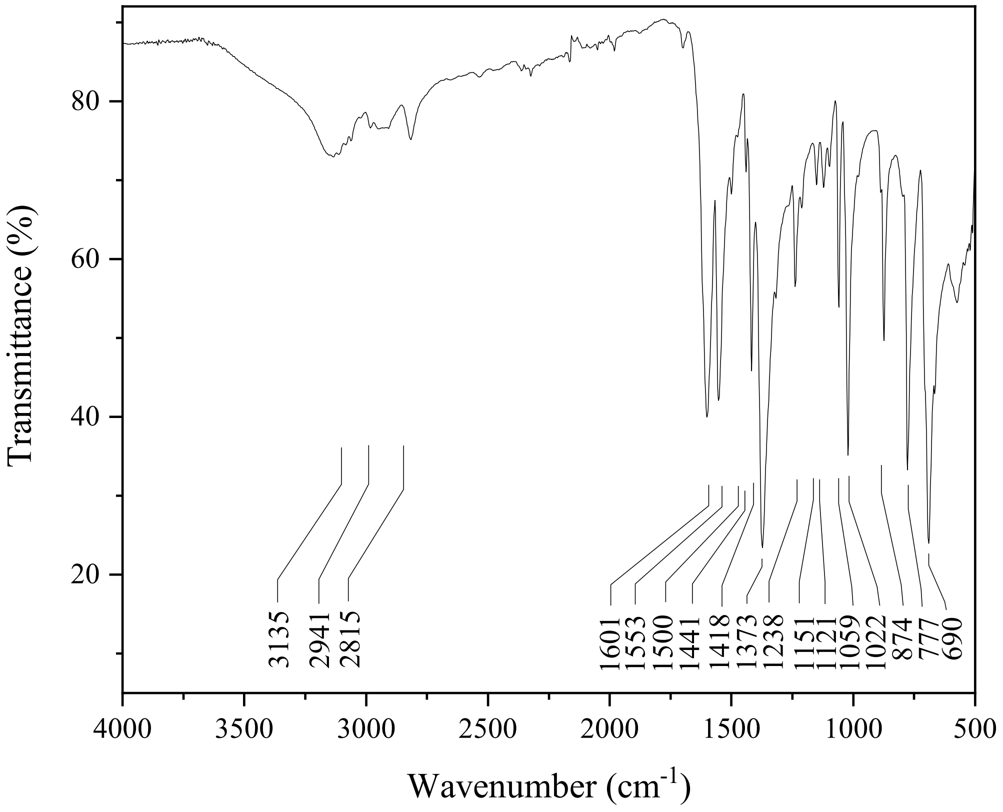

Figure S3. FTIR-ATR spectrum of compound $[Cu(ina)_2(H_2O)]_n$
